# Neurodegenerative Diseases and Cholesterol: Seeing the Field Through the Players

**DOI:** 10.3389/fnagi.2021.766587

**Published:** 2021-11-03

**Authors:** Frank W. Pfrieger

**Affiliations:** Centre National de la Recherche Scientifique, Université de Strasbourg, Institut des Neurosciences Cellulaires et Intégratives, Strasbourg, France

**Keywords:** neurologic disease, bibliometric analyses, scientific impact, sterol, research evaluation, informetric, scientometric, key opinion leader

## Abstract

Neurodegenerative diseases, namely Alzheimer’s (AD), Parkinson’s (PD), and Huntington’s disease (HD) together with amyotrophic lateral sclerosis (ALS) and multiple sclerosis (MS), devastate millions of lives per year worldwide and impose an increasing socio-economic burden across nations. Consequently, these diseases occupy a considerable portion of biomedical research aiming to understand mechanisms of neurodegeneration and to develop efficient treatments. A potential culprit is cholesterol serving as an essential component of cellular membranes, as a cofactor of signaling pathways, and as a precursor for oxysterols and hormones. This article uncovers the workforce studying research on neurodegeneration and cholesterol using the TeamTree analysis. This new bibliometric approach reveals the history and dynamics of the teams and exposes key players based on citation-independent metrics. The team-centered view reveals the players on an important field of biomedical research.

## Introduction

Neurodegenerative disorders devastate millions of lives worldwide and impose an increasing socio-economic burden (Kalia and Lang, 2015; Feigin et al., 2017; Erkkinen et al., 2018; El-Hayek et al., 2019). Research within the last decades has helped to clarify the mechanisms underlying each disease and suggested new therapeutic approaches (Fu et al., 2018; Ga et al., 2018; Jucker and Walker, 2018; Reich et al., 2018; Lassmann, 2019; Savelieff et al., 2019; Schwartz et al., 2021). A decisive step is the identification of molecular culprits that provoke or contribute to the dysfunction and degeneration of neurons. In the case of AD, research focused on three targets: hyperphosphorylated forms of tau protein, proteolytic fragments of amyloid precursor protein, and specific variants of apolipoprotein E (Long and Holtzman, 2019). A prime target for PD-related research has been alpha synuclein (Rocha et al., 2018), but other genes, as well as environmental factors, have come under scrutiny (Deng et al., 2018; Bandres-Ciga et al., 2020; Blauwendraat et al., 2020). In the case of amyotrophic lateral sclerosis (ALS), superoxide dismutase 1 has been investigated intensely as it was the first gene shown to be mutated in familial forms of the disease (Rosen et al., 1993). TAR DNA binding protein-43 (TDP-43) has become a target for ALS- and frontotemporal dementia-related research, as it was identified as a major component of ubiquitin-positive inclusions (Neumann et al., 2006). Since then, other genes have come under study as disease-causing alleles were identified in familial forms of ALS (Chia et al., 2018; Mejzini et al., 2019). Huntingtin has been at the center of attention as the long-sought gene bearing Huntington’s disease (HD)-causing mutations (The Huntington’s Disease Collaborative Research Group, 1993). Repeat expansions similar to those induced by the Huntingtin alleles cause neurodegeneration in numerous diseases including ALS and frontotemporal dementia by combinations of distinct molecular mechanisms (Malik et al., 2021; Schwartz et al., 2021). Research on multiple sclerosis (MS) has focused on immune and glial cells since chronic inflammation and demyelination are known pathologic changes preceding neurodegeneration (Faissner et al., 2019; Lassmann, 2019; Voet et al., 2019).

Why should cholesterol play a role in these diseases? Cholesterol is one of the most widely known and most studied biological molecules due to its involvement in cardiovascular and other diseases (Goldstein and Brown, 2015; Tall and Yvan-Charvet, 2015; Gliozzi et al., 2021) and due to its functions as a component of membranes in eukaryotic cells (Yeagle, 1985), as a cofactor of signaling pathways and as a precursor for steroid hormones (Miller and Auchus, 2011; Prabhu et al., 2016). Notably, cholesterol is also converted to biologically active oxysterols by specific enzymes or by autoxidation (Mutemberezi et al., 2016; Wang et al., 2021). Given the diverse functions of cholesterol, its cellular homeostasis relies on a multitude of proteins and mechanisms (Ikonen, 2008; Luo et al., 2020). In the brain, cholesterol represents a major building block due to the diversity and sheer mass of membraneous structures. This includes highly branched axons and dendrites of neurons (Elston and Fujita, 2014), fine perisynaptic processes of astrocytes (Oberheim et al., 2009), countless synaptic vesicles (Binotti et al., 2021), and the multi-layered myelin sheaths surrounding axons (Schmitt et al., 2015). Based on these considerations, disturbances of cholesterol homeostasis seem likely to cause neuronal dysfunction and degeneration. The mechanisms of cholesterol homeostasis in brain cells are probably distinct from those operating in the rest of the body (Dietschy, 2009; Pfrieger and Ungerer, 2011; Zhang and Liu, 2015; Mahley, 2016; Moutinho et al., 2016; Yoon et al., 2016; Hussain et al., 2019). Possible implications of cholesterol and derived molecules in neurodegenerative diseases have been reviewed elsewhere (Martín et al., 2014; Zarrouk et al., 2014; Leoni and Caccia, 2015; Doria et al., 2016; Arenas et al., 2017; Chang et al., 2017; Testa et al., 2018; Zarrouk et al., 2018; Adorni et al., 2019; Griffiths and Wang, 2019; Hussain et al., 2019; Jeong et al., 2019; Jin et al., 2019; Loera-Valencia et al., 2019; Petrov and Pikuleva, 2019; Segatto et al., 2019; Blauwendraat et al., 2020; González-Guevara et al., 2020; McFarlane and Kędziora-Kornatowska, 2020; Sáiz-Vazquez et al., 2020; Dai et al., 2021; Duong et al., 2021; Feringa and van der Kant, 2021; García-Sanz et al., 2021; Pikuleva and Cartier, 2021; Samant and Gupta, 2021). This article shows the workforce driving research in the field using original research articles obtained from MEDLINE (Table 1) and a new bibliometric approach (Pfrieger, 2021; https://github.com/fw-pfrieger/TeamTree). Bibliometric analyses of other aspects can be found elsewhere (Guido et al., 2015; Barboza and Ghisi, 2018; Zhang et al., 2020; Du et al., 2021; Li et al., 2021; Rizzi et al., 2021). Articles related to Niemann-Pick type C disease were excluded from the analysis as this rare lysosomal storage disorder is directly linked to perturbed cholesterol transport (Loftus et al., 1997; Naureckiene et al., 2000; Vanier, 2010).

**Table 1:** Query terms used for the literature search in PubMed/MEDLINE.

Query term*	Article count
(Q1 AND Q2) NOT Q3	4,775
(Alzheimer*[tiab]) AND Q2) NOT Q3	2,514
(Multiple sclerosis[tiab] AND Q2) NOT Q3	570
(Parkinson*[tiab] AND Q2) NOT Q3	459
((Lou Gehrig* disease[tiab] OR amyotrophic lateral sclerosis[tiab]) AND Q2) NOT Q3	132
(Huntington*[tiab] AND Q2) NOT Q3	116

*Query term 1 (Q1): ((Pick’s disease[tiab] OR progressive supranuclear palsy[tiab] OR tauopathy[tiab] OR tauopathies[tiab] OR neuronal ceroid lipofuscinosis[tiab] OR hereditary spastic paraplegia[tiab] OR ataxia telangiectasia[tiab] OR creutzfeldt-jacob[tiab] OR prion disease[tiab] OR frontotemporal dementia[tiab] OR fronto-temporal dementia[tiab] OR polyglutamine disease[tiab] OR spinocerebellar ataxia*[tiab] OR spino-cerebellar ataxia*[tiab] OR motor neurone disease[tiab] OR motor neuron disease[tiab] OR motoneuron disease[tiab] OR Lou Gehrig* disease[tiab] OR amyotrophic lateral sclerosis[tiab] OR huntington*[tiab] OR parkinson*[tiab] OR alzheimer*[tiab] OR neurodegenerative[tiab] OR neurodegeneration[tiab] OR spinal muscular atrophy[tiab] OR multiple system atrophy[tiab] OR multiple sclerosis[tiab] OR dementia[tiab]). Query term 2 (Q2): (sterol OR cholesterol OR hydroxycholesterol OR hydroxy-cholesterol OR oxysterol). Query term 3 (Q3): (review OR niemann-pick disease type c2[tiab] OR niemann-pick type c2[tiab] OR niemann-pick disease type c1[tiab] OR niemann-pick type c1[tiab] OR niemann-pick type c[tiab] OR niemann-pick disease type c[tiab]).

## Development of The Workforce Contributing to The Field

**Figure 1:**
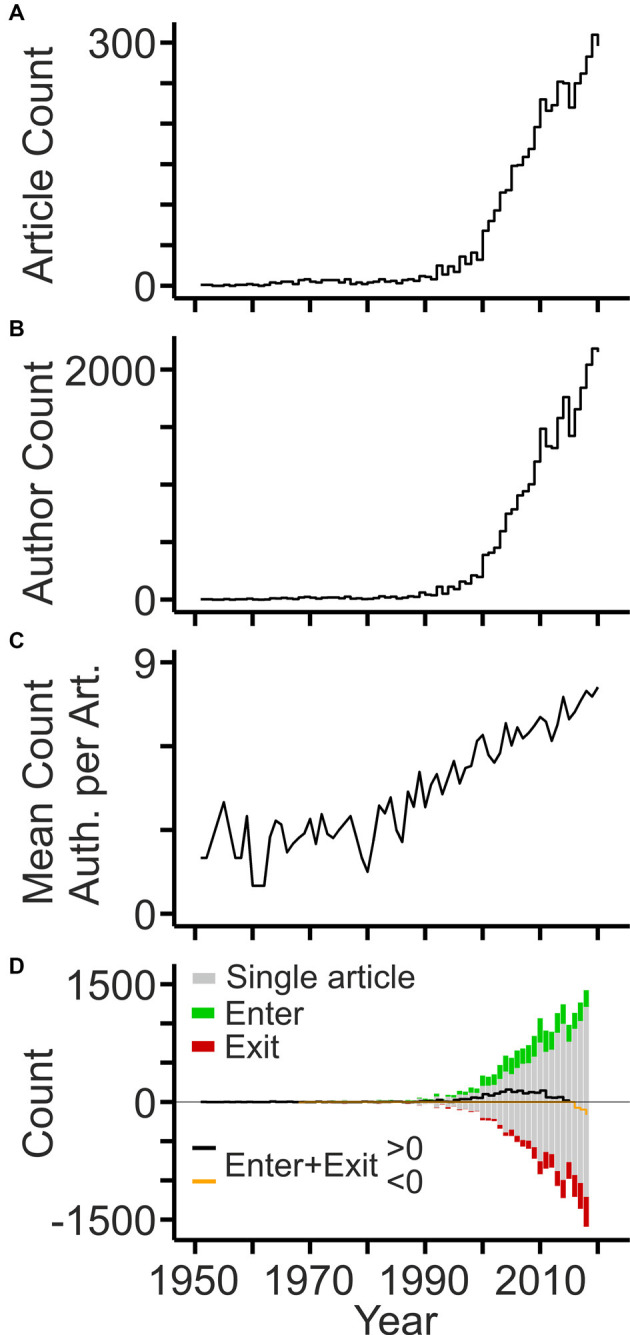
Development of the workforce. **(A)** Annual counts of original articles related to cholesterol and neurodegeneration (PubMed query shown in Table 1). **(B)** Annual counts of authors contributing to the field per year. **(C)** Mean number of authors listed on article bylines per year. **(D)** Annual counts of authors entering (green bars) and exiting (red bars) the field per year based on the first and last year of publication, respectively. Black and orange lines indicate the sum of annual author counts. Gray bars indicate the number of authors contributing single articles to the field (shown as negative and positive values).

The earliest publications date back to the 1950s when three groups investigated the cholesterol content in tissues and body fluids of patients with dementia (Mori and Barucci, 1951; Scanu et al., 1955) and MS (Chiavacci and Sperry, 1952; Poser and Curran, 1958). The number of articles published per year remained relatively low until the 1990s and increased thereafter. Since 2000, the annual count of articles has grown linearly reaching around 300 articles per year in 2020 (Figure 1A). The number of authors listed on the article byline grew in parallel, however at a much stronger pace reaching more than 2,000 per year within the last years (Figure 1B). The strong expansion of the workforce was due to an increasing number of authors per article (Figure 1C). Notably, the expansion of the field was mainly driven by authors contributing single articles, as their number grew steadily. The balance of authors publishing in the field for more than 1 year has become negative within the last years, but the number of authors leaving the field within the last years is inherently inaccurate (Figure 1D).

## Publication Records, Family Relations, and Collaborative Connections in The Field

**Figure 2:**
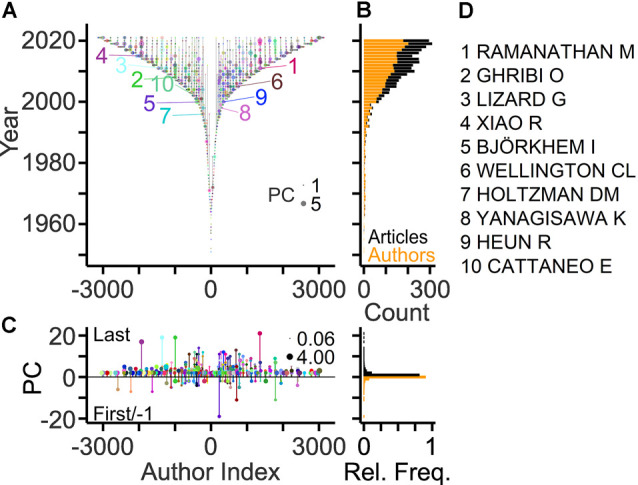
Publication records of last authors. **(A)** TeamTree graph showing the publication records of the last authors contributing to the field. Circles connected by vertical gray lines represent for each author the years of publications as the last author plotted against a chronologic author index with alternating signs and author-specific colors to enhance visibility. Circle area indicates publication count (PC) per year. Numbers indicate authors with 10 largest PCs (names indicated in panel **D**). **(B)** Number of authors entering the field per year (orange) and of articles (black) published per year. **(C)** Left, PCs per author indicating last and first author articles by positive and negative values, respectively. Circle area indicates the average number of publications per year. Right, relative frequency distributions of PC values shown on the left. **(D)** Names of authors with largest PCs in the field.

More information about the workforce can be drawn by analyzing the authors on specific positions of the article byline, which indicate the roles and contributions of authors (Claxton, 2005; Marušić et al., 2011). A total of ~3,100 authors was listed on the last byline position of articles identifying these authors as principal investigators in the field. This corresponds to 10% of the total workforce. The development of the field with respect to these contributors is shown in Figure 2A using TeamTree graphs. In this type of scatterplot, the years of publication are plotted against a chronologic index assigned to each author (Pfrieger, 2021). The number of last authors entering the field per year has grown steadily during the last two decades (Figure 2B). The total publication counts of individual last authors reached up to 21 articles, but the large majority (81%) contributed single articles (Figure 2C) as observed for the entire workforce (Figure 1D). Ranking authors by PCs identified the top contributors among the last authors (Figure 2D).

**Figure 3:**
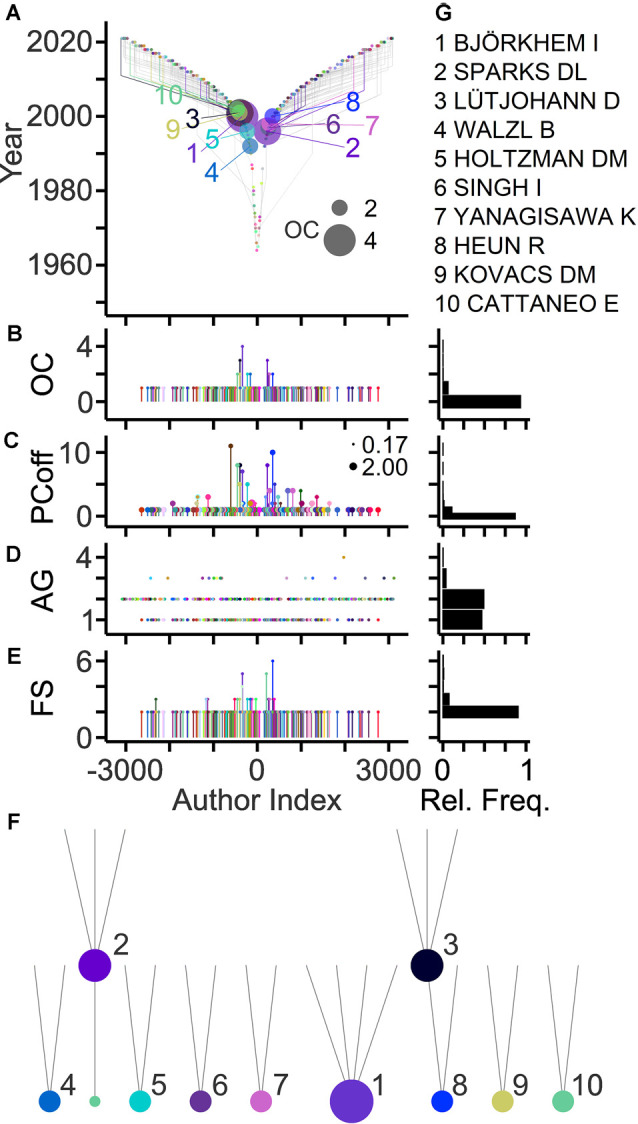
Genealogic relations in the field. **(A)** TeamTree graph showing genealogic relations among authors. Circles and gray lines indicate ancestor-offspring connections based on first author-last author pairs on article bylines. Connections of authors with the 10 largest offspring count (OC) values are shown in color (names indicated in panel **G**). Circle area indicates OC value. The signs of author indices of offspring and of ancestors were adjusted to the first-generation ancestor. **(B–E)** Quantitative data showing for individual last authors, the number of offspring **(B)** the number of articles published together with offspring (PCoff; circle area indicates average PC per year) **(C)**, the generation of authors (AG) starting with AG = 1 for first ancestors **(D)** and the family size (FS) of individual first-generation ancestors comprising all offspring across subsequent generations **(E)**. **(F,G)** Family trees **(F)** and names **(G)** of authors with 10 largest OC values (indicated by circle area).

Genealogical relations in a field can be derived from the last and first authors on article bylines representing ancestor and offspring, respectively (Pfrieger, 2021). Figure 3A shows family relations among authors highlighting those with the largest offspring counts. About 10% of last authors published previously as first authors thus qualifying as offspring, and 7% of last authors qualified as ancestors (Figure 3B). These ancestors generated up to four offspring authors and published up to 10 articles with their offspring (Figure 3C). Overall, the field comprised 192 families with up to six members spanning maximally four generations (Figures 3D,E). The large majority of families (91%) had only two members. Ranking by OCs revealed the most prolific authors and their families in the field (Figures 3F,G).

**Figure 4:**
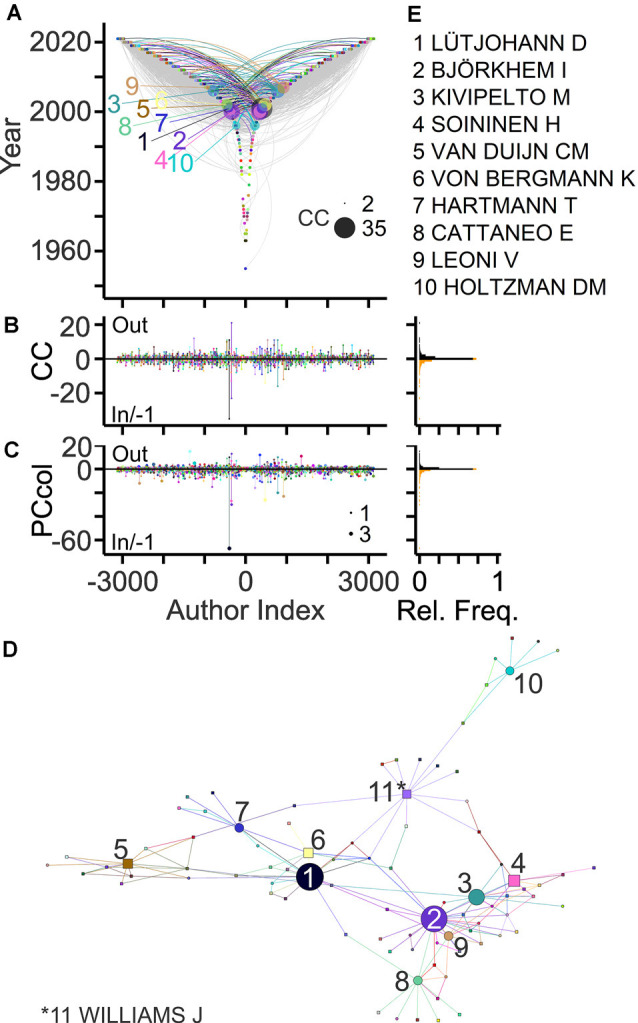
Collaborative connections in the field. **(A)** TeamTree graph showing collaborations between last authors (out; negative author index) and (non-first) co-authors (in; positive author index) on article bylines. For out- and in-degree connections an author lists other authors as co-authors and an author is listed as a co-author, respectively. Connections of authors with 10 highest connection count (CC) values (in+out) are shown in color (names indicated in panel **E**). Circle areas indicate CCout and CCin values of these authors. **(B,C)** Left, counts of collaborators **(B)** and of collaborative articles **(C)** per author. Circle area indicates PCannu. Right, relative frequency distributions of parameters shown on the left. **(D,E)** Network **(D)** and names **(E)** of authors with 10 largest CC values. Symbol areas **(D)** indicate CC values normalized to the maximum. Circles and rectangles represent family and non-family authors, respectively.

Collaborative connections can be delineated based on middle and last byline positions (Newman, 2001; Pfrieger, 2021). Figure 4 exposes collaborations between authors contributing to the field. In total, 43% of the authors established collaborations with maximally 46 other authors and published up to 77 collaborative articles as last and co-author, respectively (Figures 4B,C). Ranking authors based on collaboration counts revealed the most strongly connected teams in the field and their networks (Figures 4D,E).

## Identification of Major Contributors to The Field

**Figure 5:**
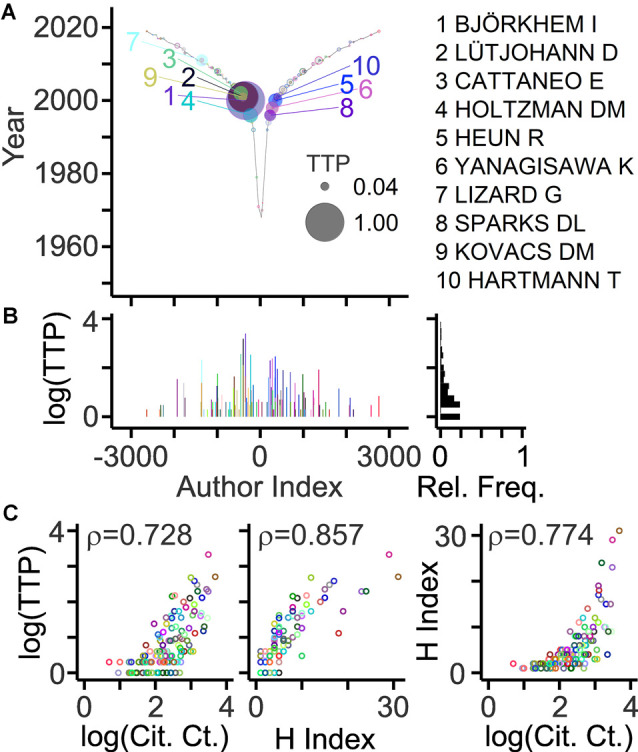
Author ranking based on the TeamTree product. **(A)** Graph showing the TeamTree product (TTP) of individual last authors in the field represented by their author indices. This new metric takes into account publication records, offspring training and mentorship, and collaborative connections. Numerically, it represents the product of PC (last author articles) × OC × CC. Circle sizes indicate TTP values normalized to the maximum. Colored circles and numbers indicate authors with 10 highest values. Their names are shown on the right. Gray circles with colored border indicate authors with TTP values above zero. **(B)** Log10(TTP) values and their relative frequency distribution. **(C)** Scatterplots with circles representing individual authors (indicated by color; different from panels **A,B**) with their TTP values (log10) plotted against the total number of citing articles (left; Cit. Ct.; log10 values) and their H indices (middle) and with their H indices plotted against the total number of citing articles (right; log10 values). Numbers represent correlation coefficients [Spearman’s rho values; two-sided test; *n* = 126; *S* = 90,803 (left)/47,558 (middle)/75,414 (right); *p* < 10^−10^]. Citation-related parameters were calculated from bibliographic records obtained by a Web of Science query (Clarivate Analytics).

An important goal of bibliometric analyses is to estimate the contribution of individual authors. The “key players” may serve as experts, key opinion leaders, referees, and collaborators. Different indicators of scientific production have been explored including PCs, citations, invitations, grants, and honors (Hicks et al., 2015; Schimanski and Alperin, 2018; Braithwaite et al., 2019). Original articles represent an accessible primary basis to estimate the contribution of an author. A new approach takes into account publication record, offspring generation, and collaborative connections, and delivers a new citation-independent parameter named TeamTree product (TTP; Pfrieger, 2021). Based on this parameter, key players studying neurodegenerative diseases and cholesterol are exposed in Figure 5. Due to the high selectivity, only a small fraction of authors (5%) reached TTP values above zero. Notably, TTP values of authors were strongly correlated with citation-dependent measures such as the total number of citations or the H index (Figure 5C).

## Disease-Specific Workforce Analyses

**Figure 6:**
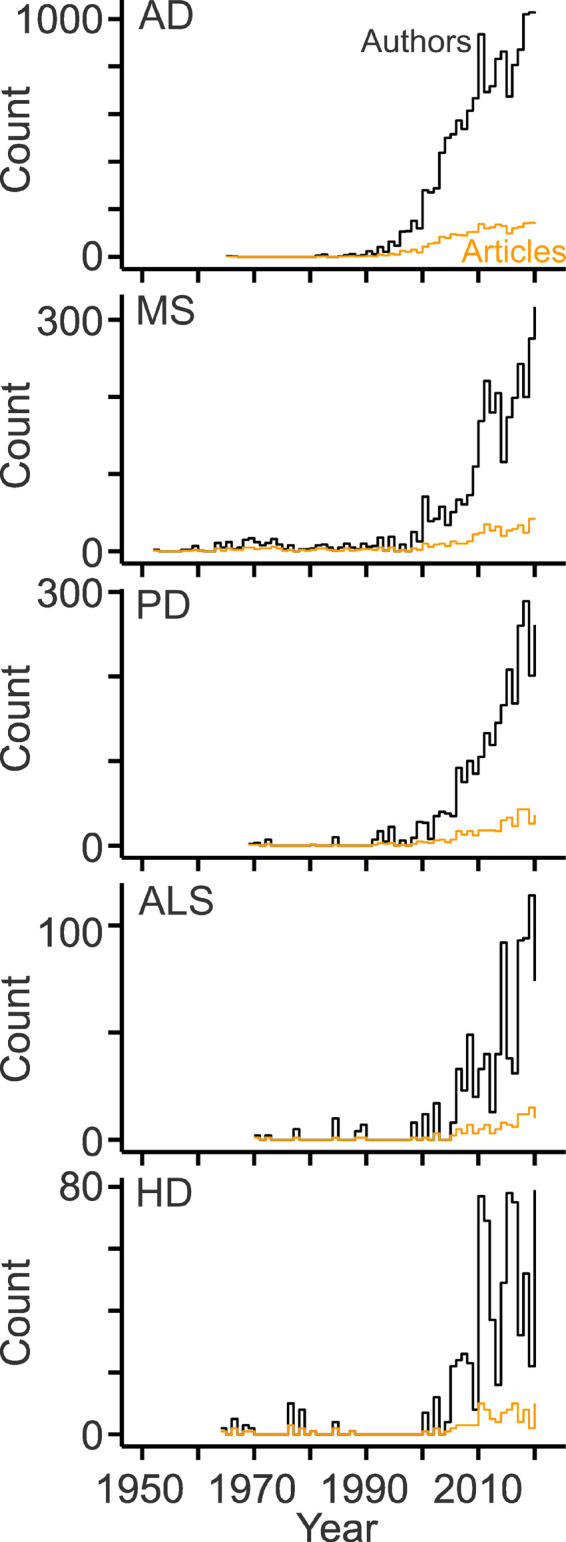
Development of the disease-specific workforce. Line plots showing counts of original articles (orange) and of the contributing authors (black) per year related to cholesterol and the indicated diseases. AD, Alzheimer’s disease; MS, multiple sclerosis; PD, Parkinson’s disease; ALS, amyotrophic lateral sclerosis; HD, Huntington’s disease.

To gain deeper insight, diseases with the largest numbers of publications were analyzed separately (Table 1). Notably, AD-related research produced half of the articles published in the field (Table 1). Overall, the fields showed marked differences with respect to length and growth pattern: MS has the longest and most continuous publication record (Figure 6). Except for two articles published in the 1960s, research on AD and cholesterol started in the 1980s. The subsequent growth of this field was probably triggered by discoveries that the epsilon allele of apolipoprotein E (Corder et al., 1993; Poirier et al., 1993; Rebeck et al., 1993; Saunders et al., 1993; Strittmatter et al., 1993) and high blood levels of cholesterol raise the risk of sporadic AD (Kivipelto et al., 2001). Parallel studies revealed connections between cholesterol and beta amyloid (Hartmann et al., 1994; Bodovitz and Klein, 1996; Avdulov et al., 1997; Howland et al., 1998; Simons et al., 1998; Refolo et al., 2000; Fassbender et al., 2001; Kojro et al., 2001; Puglielli et al., 2001; Runz et al., 2002; Wahrle et al., 2002) and between statins and AD (Wolozin et al., 2000; Refolo et al., 2001). The other disease fields are characterized by intermittent publication activity starting in the 1960s (HD) and 1970 (PD, ALS) and a more continuous development since 2000 (Figure 6). In the case of HD, pioneering studies showing links to cholesterol synthesis were published at the beginning of the 2000s (Sipione et al., 2002; Valenza et al., 2005). In all fields, the workforce grew more strongly than the number of publications (Figure 6) due to the increasing number of authors per article (Figure 1C). The ratios of author counts to publication counts were very similar across fields (6.6 ± 0.5; mean ± standard deviation; *n* = 5).

**Figure 7:**
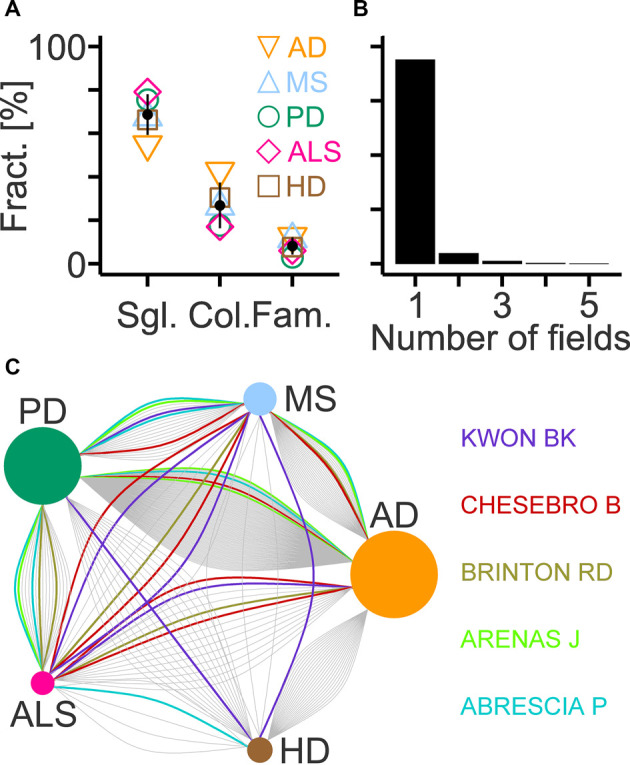
Workforce composition and overlap across selected diseases. **(A)** Fractions of authors contributing single articles compared to the total workforce (Sgl.), of collaborating authors among last authors (Col.) and of authors with family ties among last authors (Fam.) in indicated fields (AD, Alzheimer’s disease; MS, multiple sclerosis; PD, Parkinson’s disease; ALS, amyotrophic lateral sclerosis; HD, Huntington’s disease). Black circles and lines indicate mean and standard deviation (*n* = 5), respectively. **(B)** Histogram showing the fraction of last authors that contributed articles to the indicated number of fields. **(C)** Diagram showing connections between two diseases that are established by last authors contributing to both fields. Names and colored lines indicate the last authors with the highest number of connections (*n* = 6). Circle size represents the number of connections normalized to the maximum (AD; 160 links).

In each field, most authors contributed single articles with their fractions ranging from the lowest value in AD to the highest in ALS (Figure 7A). Inversely, the AD and ALS fields showed the highest and lowest fraction of authors involved in collaborations, respectively (Figure 7A). Authors with family ties represented a minority of the workforce with disease-specific fractions between 3% and 13% (Figure 7A). The analysis also revealed relatively little overlap among the workforce of each disease. Only 6% of authors (146 out of 2,379) contributed articles to more than one field (Figure 7B) and established up to six connections among them with AD and PD showing the largest workforce overlap (Figure 7C).

**Figure 8:**
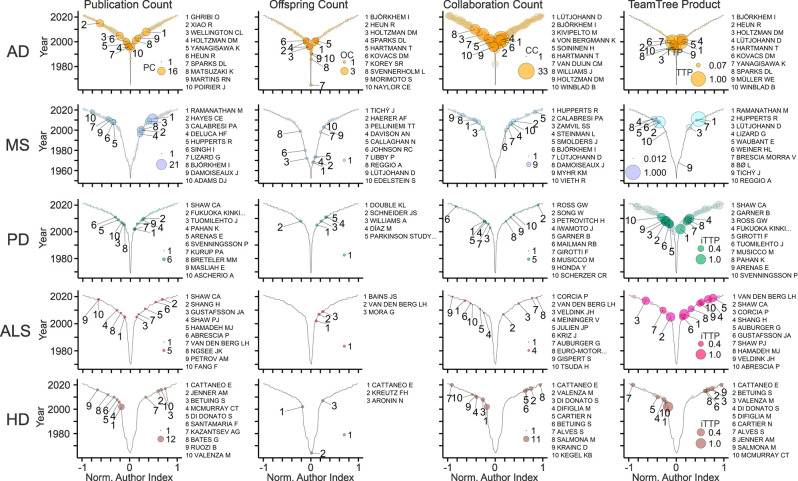
In-depth view on the field-specific workforce. TeamTree graphs showing counts of publications (PC), offspring (OC), collaborative connections (CC), and the TeamTree product (TTP) in the indicated fields (AD, Alzheimer’s disease; MS, multiple sclerosis; PD, Parkinson’s disease; ALS, amyotrophic lateral sclerosis; HD, Huntington’s disease) together with names of authors with the 10 largest values for each parameter. Note that for PD, ALS and HD, TTP values were replaced by an inclusive version of this measure (iTTP). For iTTP, zero counts of OC or CC values are set to one to include authors without offspring or lacking collaborators in the TTP-based ranking.

TeamTree graphs illustrate the workforce that studies links between cholesterol and the selected diseases (Table 1; Figure 8). Not surprisingly key players of the AD field dominate the global rankings (Figures 2–5, 8). The analysis shows further that OCs are particularly sensitive to the size of the field. In those with the lowest number of articles and the smallest workforce (PD, ALS, HD), authors produced maximally one offspring indicating that this parameter requires a critical mass of authors (Figure 8). The TTP values reveal distinct disease-specific origins of the top 10 contributors. Notably, in the AD field, these authors entered the field within one decade starting in the 1990s, whereas, in other fields, these contributors entered after the year 2000 (Figure 8).

## Conclusions

The new bibliometric analysis provides a detailed view of the development and structure of the workforce driving research on cholesterol and neurodegenerative diseases and complements content-specific summaries. The analysis revealed that the field started in the 1950s and remained relatively small until the 1990s. Except for MS, all fields showed intermittent publications, but a strong growth since 2000. The continuous expansion of the workforce during this period was mainly driven by authors contributing single articles although their contribution varied among the diseases analyzed. More than half of the articles are related to AD, therefore, the family ties, collaborative connections, and key players of this field dominate the overall picture. The analysis has caveats. A key challenge for this and other bibliometric approaches are ambiguous author names, as distinct authors can share the same name precluding correct evaluation (Smalheiser and Torvik, 2009). Evaluation of contributions based on single metrics such as TTP values is context-dependent, unsuited to evaluate junior scientists, and insensitive to ground-breaking contributions from small teams or from teams that contribute only briefly to a field.

## Author Contributions

FWP designed the review, performed literature queries, wrote and validated the code, analyzed the bibliographic records, prepared figures, and wrote the manuscript.

## Conflict of Interest

The author declares that the research was conducted in the absence of any commercial or financial relationships that could be construed as a potential conflict of interest.

## Publisher’s Note

All claims expressed in this article are solely those of the authors and do not necessarily represent those of their affiliated organizations, or those of the publisher, the editors and the reviewers. Any product that may be evaluated in this article, or claim that may be made by its manufacturer, is not guaranteed or endorsed by the publisher.

## References

[B1] Adorni M. P. Ruscica M. Ferri N. Bernini F. Zimetti F. (2019). Proprotein convertase subtilisin/kexin type 9, brain cholesterol homeostasis and potential implication for Alzheimer’s disease. Front. Aging Neurosci. 11:120. 10.3389/fnagi.2019.00120 31178716 PMC6538876

[B2] Arenas F. Garcia-Ruiz C. Fernandez-Checa J. C. (2017). Intracellular cholesterol trafficking and impact in neurodegeneration. Front. Mol. Neurosci. 10:382. 10.3389/fnmol.2017.00382 29204109 PMC5698305

[B3] Avdulov N. A. Chochina S. V. Igbavboa U. Warden C. S. Vassiliev A. V. Wood W. G. (1997). Lipid binding to amyloid β-peptide aggregates: preferential binding of cholesterol as compared with phosphatidylcholine and fatty acids. J. Neurochem. 69, 1746–1752. 10.1046/j.1471-4159.1997.69041746.x 9326304

[B4] Bandres-Ciga S. Diez-Fairen M. Kim J. J. Singleton A. B. (2020). Genetics of Parkinson’s disease: an introspection of its journey towards precision medicine. Neurobiol. Dis. 137:104782. 10.1016/j.nbd.2020.104782 31991247 PMC7064061

[B5] Barboza L. A. Ghisi N. C. (2018). Evaluating the current state of the art of Huntington disease research: a scientometric analysis. Braz. J. Med. Biol. Res. 51:e6299. 10.1590/1414-431X20176299 29340519 PMC5769753

[B6] Binotti B. Jahn R. Pérez-Lara Á. (2021). An overview of the synaptic vesicle lipid composition. Arch. Biochem. Biophys. 709:108966. 10.1016/j.abb.2021.108966 34139199

[B7] Blauwendraat C. Nalls M. A. Singleton A. B. (2020). The genetic architecture of Parkinson’s disease. Lancet Neurol. 19, 170–178. 10.1016/S1474-4422(19)30287-X 31521533 PMC8972299

[B8] Bodovitz S. Klein W. L. (1996). Cholesterol modulates α-secretase cleavage of amyloid precursor protein. J. Biol. Chem. 271, 4436–4440. 10.1074/jbc.271.8.4436 8626795

[B9] Braithwaite J. Herkes J. Churruca K. Long J. C. Pomare C. Boyling C.. (2019). Comprehensive Researcher Achievement Model (CRAM): a framework for measuring researcher achievement, impact and influence derived from a systematic literature review of metrics and models. BMJ Open 9:e025320. 10.1136/bmjopen-2018-025320 30928941 PMC6475357

[B10] Chang T. Y. Yamauchi Y. Hasan M. T. Chang C. (2017). Cellular cholesterol homeostasis and Alzheimer’s disease. J. Lipid Res. 58, 2239–2254. 10.1194/jlr.R075630 28298292 PMC5711498

[B11] Chia R. Chiò A. Traynor B. J. (2018). Novel genes associated with amyotrophic lateral sclerosis: diagnostic and clinical implications. Lancet Neurol. 17, 94–102. 10.1016/S1474-4422(17)30401-5 29154141 PMC5901717

[B12] Chiavacci L. V. Sperry W. M. (1952). Concentration of cholesterol and of lipid phosphorus in blood serum in multiple sclerosis. AMA Arch. Neurol. Psychiatry 68, 37–42. 10.1001/archneurpsyc.1952.02320190043003 14932544

[B13] Claxton L. D. (2005). Scientific authorship: part 2. History, recurring issues, practices, and guidelines. Mutat. Res. 589, 31–45. 10.1016/j.mrrev.2004.07.002 15652225

[B14] Corder E. H. Saunders A. M. Strittmatter W. J. Schmechel D. E. Gaskell P. C. Small G. W.. (1993). Gene dose of apolipoprotein E type 4 allele and the risk of Alzheimer’s disease in late onset families. Science 261, 921–923. 10.1126/science.8346443 8346443

[B15] Dai L. Zou L. Meng L. Qiang G. Yan M. Zhang Z. (2021). Cholesterol metabolism in neurodegenerative diseases: molecular mechanisms and therapeutic targets. Mol. Neurobiol. 58, 2183–2201. 10.1007/s12035-020-02232-6 33411241

[B16] Deng H. Wang P. Jankovic J. (2018). The genetics of Parkinson disease. Ageing Res. Rev. 42, 72–85. 10.1016/j.arr.2017.12.007 29288112

[B17] Dietschy J. M. (2009). Central nervous system: cholesterol turnover, brain development and neurodegeneration. Biol. Chem. 390, 287–293. 10.1515/BC.2009.035 19166320 PMC3066069

[B18] Doria M. Maugest L. Moreau T. Lizard G. Vejux A. (2016). Contribution of cholesterol and oxysterols to the pathophysiology of Parkinson’s disease. Free Radic. Biol. Med. 101, 393–400. 10.1016/j.freeradbiomed.2016.10.008 27836779

[B19] Du Y.-H. Yang R.-Y. Wang Q. Wang L.-Y. Liang L.-C. Zhu L.. (2021). Bibliometric analysis study on the mechanisms of brain energy metabolism disorders in Alzheimer’s disease from 2000 to 2020. Front. Neurol. 12:670220. 10.3389/fneur.2021.670220 34354657 PMC8333709

[B20] Duong M. T. Nasrallah I. M. Wolk D. A. Chang C. C. Y. Chang T. Y. (2021). Cholesterol, atherosclerosis, and APOE in vascular contributions to cognitive impairment and dementia (VCID): potential mechanisms and therapy. Front. Aging Neurosci. 13:647990. 10.3389/fnagi.2021.647990 33841127 PMC8026881

[B21] El-Hayek Y. H. Wiley R. E. Khoury C. P. Daya R. P. Ballard C. Evans A. R.. (2019). Tip of the iceberg: assessing the global socioeconomic costs of Alzheimer’s disease and related dementias and strategic implications for stakeholders. J. Alzheimers Dis. 70, 323–341. 10.3233/JAD-190426 31256142 PMC6700654

[B22] Elston G. N. Fujita I. (2014). Pyramidal cell development: postnatal spinogenesis, dendritic growth, axon growth, and electrophysiology. Front. Neuroanat. 8:78. 10.3389/fnana.2014.00078 25161611 PMC4130200

[B23] Erkkinen M. G. Kim M. O. Geschwind M. D. (2018). Clinical neurology and epidemiology of the major neurodegenerative diseases. Cold Spring Harb. Perspect. Biol. 10:a033118. 10.1101/cshperspect.a033118 28716886 PMC5880171

[B24] Faissner S. Plemel J. R. Gold R. Yong V. W. (2019). Progressive multiple sclerosis: from pathophysiology to therapeutic strategies. Nat. Rev. Drug Discov. 18, 905–922. 10.1038/s41573-019-0035-2 31399729

[B25] Fassbender K. Simons M. Bergmann C. Stroick M. Lutjohann D. Keller P.. (2001). Simvastatin strongly reduces levels of Alzheimer’s disease β -amyloid peptides Aβ 42 and Aβ 40 *in vitro* and *in vivo*. Proc. Natl. Acad. Sci. U S A 98, 5856–5861. 10.1073/pnas.081620098 11296263 PMC33303

[B26] Feigin V. L. Abajobir A. A. Abate K. H. Abd-Allah F. Abdulle A. M. Abera S. F.. (2017). Global, regional, and national burden of neurological disorders during 1990–2015: a systematic analysis for the Global Burden of Disease Study 2015. Lancet Neurol. 16, 877–897. 10.1016/S1474-4422(17)30299-5 28931491 PMC5641502

[B27] Feringa F. M. van der Kant R. (2021). Cholesterol and Alzheimer’s disease; from risk genes to pathological effects. Front. Aging Neurosci. 13:690372. 10.3389/fnagi.2021.690372 34248607 PMC8264368

[B28] Fu H. J. Hardy J. Duff K. E. (2018). Selective vulnerability in neurodegenerative diseases. Nat. Neurosci. 21, 1350–1358. 10.1038/s41593-018-0221-2 30250262 PMC6360529

[B29] Ga L. Cookson M. R. Petrucelli L. La Spade A. R. (2018). Converging pathways in neurodegeneration, from genetics to mechanisms. Nat. Neurosci. 21, 1300–1309. 10.1038/s41593-018-0237-7 30258237 PMC6278826

[B30] García-Sanz P. Aerts J. Moratalla R. (2021). The role of cholesterol in α-synuclein and lewy body pathology in GBA1 Parkinson’s disease. Mov. Disord. 36, 1070–1085. 10.1002/mds.28396 33219714 PMC8247417

[B31] Gliozzi M. Musolino V. Bosco F. Scicchitano M. Scarano F. Nucera S.. (2021). Cholesterol homeostasis: researching a dialogue between the brain and peripheral tissues. Pharmacol. Res. 163:105215. 10.1016/j.phrs.2020.105215 33007421

[B32] Goldstein J. L. Brown M. S. (2015). A century of cholesterol and coronaries: from plaques to genes to statins. Cell 161, 161–172. 10.1016/j.cell.2015.01.036 25815993 PMC4525717

[B33] González-Guevara E. Cárdenas G. Pérez-Severiano F. Martínez-Lazcano J. C. (2020). Dysregulated brain cholesterol metabolism is linked to neuroinflammation in Huntington’s disease. Mov. Disord. 35, 1113–1127. 10.1002/mds.28089 32410324

[B34] Griffiths W. J. Wang Y. (2019). Oxysterol research: a brief review. Biochem. Soc. Trans. 47, 517–526. 10.1042/BST20180135 30936243 PMC6490702

[B35] Guido D. Morandi G. Palluzzi F. Borroni B. (2015). Telling the story of frontotemporal dementia by bibliometric analysis. J. Alzheimers Dis. 48, 703–709. 10.3233/JAD-150275 26402093

[B36] Hartmann H. Eckert A. Müller W. E. (1994). Apolipoprotein E and cholesterol affect neuronal calcium signalling: the possible relationship to β-amyloid neurotoxicity. Biochem. Biophys. Res. Commun. 200, 1185–1192. 10.1006/bbrc.1994.1576 8185566

[B37] Hicks D. Wouters P. Waltman L. De Rijcke S. Rafols I. (2015). The Leiden Manifesto for research metrics. Nature 520, 429–431. 10.1038/520429a 25903611

[B38] Howland D. S. Trusko S. P. Savage M. J. Reaume A. G. Lang D. M. Hirsch J. D.. (1998). Modulation of secreted β-amyloid precursor protein and amyloid β-peptide in brain by cholesterol. J. Biol. Chem. 273, 16576–16582. 10.1074/jbc.273.26.16576 9632729

[B39] Hussain G. Wang J. Rasul A. Anwar H. Imran A. Qasim M.. (2019). Role of cholesterol and sphingolipids in brain development and neurological diseases. Lipids Health Dis. 18:26. 10.1186/s12944-019-0965-z 30683111 PMC6347843

[B40] Ikonen E. (2008). Cellular cholesterol trafficking and compartmentalization. Nat. Rev. Mol. Cell Biol. 9, 125–138. 10.1038/nrm2336 18216769

[B41] Jeong W. Lee H. Cho S. Seo J. (2019). ApoE4-induced cholesterol dysregulation and its brain cell type-specific implications in the pathogenesis of Alzheimer’s disease. Mol. Cells 42, 739–746. 10.14348/molcells.2019.0200 31711277 PMC6883979

[B42] Jin U. Park S. J. Park S. M. (2019). Cholesterol metabolism in the brain and its association with Parkinson’s disease. Exp. Neurobiol. 28, 554–567. 10.5607/en.2019.28.5.554 31698548 PMC6844833

[B43] Jucker M. Walker L. C. (2018). Propagation and spread of pathogenic protein assemblies in neurodegenerative diseases. Nat. Neurosci. 21, 1341–1349. 10.1038/s41593-018-0238-6 30258241 PMC6375686

[B44] Kalia L. V. Lang A. E. (2015). Parkinson’s disease. Lancet 386, 896–912. 10.1016/S0140-6736(14)61393-3 25904081

[B45] Kivipelto M. Helkala E. L. Laakso M. P. Hanninen T. Hallikainen M. Alhainen K.. (2001). Midlife vascular risk factors and Alzheimer’s disease in later life: longitudinal, population based study. Br. Med. J. 322, 1447–1451. 10.1136/bmj.322.7300.1447 11408299 PMC32306

[B46] Kojro E. Gimpl G. Lammich S. Marz W. Fahrenholz F. (2001). Low cholesterol stimulates the nonamyloidogenic pathway by its effect on the α -secretase ADAM 10. Proc. Natl. Acad. Sci. U S A 98, 5815–5820. 10.1073/pnas.081612998 11309494 PMC33296

[B47] Lassmann H. (2019). Pathogenic mechanisms associated with different clinical courses of multiple sclerosis. Front. Immunol. 9:3116. 10.3389/fimmu.2018.03116 30687321 PMC6335289

[B48] Leoni V. Caccia C. (2015). The impairment of cholesterol metabolism in Huntington disease. Biochim. Biophys. Acta 1851, 1095–1105. 10.1016/j.bbalip.2014.12.018 25596342

[B49] Li Y. Fang R. Liu Z. Jiang L. Zhang J. Li H.. (2021). The association between toxic pesticide environmental exposure and Alzheimer’s disease: a scientometric and visualization analysis. Chemosphere 263:128238. 10.1016/j.chemosphere.2020.128238 33297185

[B50] Loera-Valencia R. Goikolea J. Parrado-Fernandez C. Merino-Serrais P. Maioli S. (2019). Alterations in cholesterol metabolism as a risk factor for developing Alzheimer’s disease: potential novel targets for treatment. J. Steroid Biochem. Mol. Biol. 190, 104–114. 10.1016/j.jsbmb.2019.03.003 30878503

[B51] Loftus S. K. Morris J. A. Carstea E. D. Gu J. Z. Cummings C. Brown A.. (1997). Murine model of niemann-pick C disease: mutation in a cholesterol homeostasis gene. Science 277, 232–235. 10.1126/science.277.5323.232 9211850

[B52] Long J. M. Holtzman D. M. (2019). Alzheimer disease: an update on pathobiology and treatment strategies. Cell 179, 312–339. 10.1016/j.cell.2019.09.001 31564456 PMC6778042

[B53] Luo J. Yang H. Y. Song B.-L. (2020). Mechanisms and regulation of cholesterol homeostasis. Nat. Rev. Mol. Cell Biol. 21, 225–245. 10.1038/s41580-019-0190-7 31848472

[B54] Mahley R. W. (2016). Central nervous system lipoproteins: apoe and regulation of cholesterol metabolism. Arterioscler. Thromb. Vasc. Biol. 36, 1305–1315. 10.1161/ATVBAHA.116.307023 27174096 PMC4942259

[B55] Malik I. Kelley C. P. Wang E. T. Todd P. K. (2021). Molecular mechanisms underlying nucleotide repeat expansion disorders. Nat. Rev. Mol. Cell Biol. 22, 589–607. 10.1038/s41580-021-00382-6 34140671 PMC9612635

[B56] Martín M. G. Pfrieger F. Dotti C. G. (2014). Cholesterol in brain disease: sometimes determinant and frequently implicated. EMBO Rep. 15, 1036–1052. 10.15252/embr.201439225 25223281 PMC4253844

[B57] Marušić A. Bošnjak L. Jerončić A. (2011). A systematic review of research on the meaning, ethics and practices of authorship across scholarly disciplines. PLoS One 6:e23477. 10.1371/journal.pone.0023477 21931600 PMC3169533

[B58] McFarlane O. Kędziora-Kornatowska K. (2020). Cholesterol and dementia: a long and complicated relationship. Curr. Aging Sci. 13, 42–51. 10.2174/1874609812666190917155400 31530269 PMC7403650

[B59] Mejzini R. Flynn L. L. Pitout L. L. Fletcher S. Wilton S. D. Akkari P. A. (2019). ALS genetics, mechanisms, and therapeutics: where are we now? Front. Neurosci. 13:1310. 10.3389/fnins.2019.01310 31866818 PMC6909825

[B60] Miller W. L. Auchus R. J. (2011). The molecular biology, biochemistry ,and physiology of human steroidogenesis and its disorders. Endocr. Rev. 32, 81–151. 10.1210/er.2010-0013 21051590 PMC3365799

[B61] Mori F. Barucci M. (1951). [Cholesterol content of the adrenals in dementia]. Boll. Soc. Ital. Biol. Sper. 27, 1029–1030. 14895681

[B62] Moutinho M. Nunes M. J. Rodrigues E. (2016). Cholesterol 24-hydroxylase: brain cholesterol metabolism and beyond. Biochim. Biophys. Acta 1861, 1911–1920. 10.1016/j.bbalip.2016.09.011 27663182

[B63] Mutemberezi V. Guillemot-Legris O. Muccioli G. G. (2016). Oxysterols: from cholesterol metabolites to key mediators. Prog. Lipid Res. 64, 152–169. 10.1016/j.plipres.2016.09.002 27687912

[B64] Naureckiene S. Sleat D. Lackland H. Fensom A. Vanier M. T. Wattiaux R.. (2000). Identification of HE1 as the second gene of niemann-pick C disease. Science 290, 2298–2301. 10.1126/science.290.5500.2298 11125141

[B65] Neumann M. Sampathu D. M. Kwong L. K. Truax A. C. Micsenyi M. C. Chou T. T.. (2006). Ubiquitinated TDP-43 in frontotemporal lobar degeneration and amyotrophic lateral sclerosis. Science 314, 130–133. 10.1126/science.1134108 17023659

[B66] Newman M. E. (2001). The structure of scientific collaboration networks. Proc. Natl. Acad. Sci. U S A 98, 404–409. 10.1073/pnas.021544898 11149952 PMC14598

[B67] Oberheim N. A. Takano T. Han X. He W. Lin J. H. Wang F.. (2009). Uniquely hominid features of adult human astrocytes. J. Neurosci. 29, 3276–3287. 10.1523/JNEUROSCI.4707-08.2009 19279265 PMC2819812

[B68] Petrov A. M. Pikuleva I. A. (2019). Cholesterol 24-hydroxylation by CYP46A1: benefits of modulation for brain diseases. Neurotherapeutics 16, 635–648. 10.1007/s13311-019-00731-6 31001737 PMC6694357

[B69] Pfrieger F. W. (2021). TeamTree analysis: a new approach to evaluate scientific production. PLoS One 16:e0253847. 10.1371/journal.pone.0253847 34288914 PMC8294527

[B70] Pfrieger F. W. Ungerer N. (2011). Cholesterol metabolism in neurons and astrocytes. Prog. Lipid Res. 50, 357–371. 10.1016/j.plipres.2011.06.002 21741992

[B71] Pikuleva I. A. Cartier N. (2021). Cholesterol hydroxylating cytochrome P450 46A1: from mechanisms of action to clinical applications. Front. Aging Neurosci. 13:696778. 10.3389/fnagi.2021.696778 34305573 PMC8297829

[B72] Poirier J. Davignon J. Bouthillier D. Kogan S. Bertrand P. Gauthier S. (1993). Apolipoprotein E polymorphism and Alzheimer’s disease. Lancet 342, 697–699. 10.1016/0140-6736(93)91705-q 8103819

[B73] Poser C. M. Curran G. L. (1958). Cerebrospinal fluid free cholesterol as index of activity of multiple sclerosis and allied diseases. AMA Arch. Neurol. Psychiatry 80, 304–313. 10.1001/archneurpsyc.1958.02340090040005 13570746

[B74] Prabhu A. V. Luu W. N. Li D. F. Sharpe L. J. Brown A. J. (2016). DHCR7: a vital enzyme switch between cholesterol and vitamin D production. Prog. Lipid Res. 64, 138–151. 10.1016/j.plipres.2016.09.003 27697512

[B75] Puglielli L. Konopka G. Pack-Chung E. Ingano L. A. Berezovska O. Hyman B. T.. (2001). Acyl-coenzyme A: cholesterol acyltransferase modulates the generation of the amyloid β-peptide. Nat. Cell Biol. 3, 905–912. 10.1038/ncb1001-905 11584272

[B76] Rebeck G. W. Reiter J. S. Strickland D. K. Hyman B. T. (1993). Apolipoprotein E in sporadic Alzheimer’s disease: allelic variation and receptor interactions. Neuron 11, 575–580. 10.1016/0896-6273(93)90070-8 8398148

[B77] Refolo L. M. Pappolla M. A. Lafrancois J. Malester B. Schmidt S. D. Thomas-Bryant T.. (2001). A cholesterol-lowering drug reduces β-amyloid pathology in a transgenic mouse model of Alzheimer’s disease. Neurobiol. Dis. 8, 890–899. 10.1006/nbdi.2001.0422 11592856

[B78] Refolo L. M. Pappolla M. A. Malester B. Lafrancois J. Bryant-Thomas T. Wang R.. (2000). Hypercholesterolemia accelerates the Alzheimer’s amyloid pathology in a transgenic mouse model. Neurobiol. Dis. 7, 321–331. 10.1006/nbdi.2000.0304 10964604

[B79] Reich D. S. Lucchinetti C. F. Calabresi P. A. (2018). Multiple sclerosis. N. Engl. J. Med. 378, 169–180. 10.1056/NEJMra1401483 29320652 PMC6942519

[B80] Rizzi L. Aventurato Í. K. Balthazar M. L. F. (2021). Neuroimaging research on dementia in brazil in the last decade: scientometric analysis, challenges, and peculiarities. Front. Neurol. 12:640525. 10.3389/fneur.2021.640525 33790850 PMC8005640

[B81] Rocha E. M. De Miranda B. Sanders L. H. (2018). α-synuclein: pathology, mitochondrial dysfunction and neuroinflammation in Parkinson’s disease. Neurobiol. Dis. 109, 249–257. 10.1016/j.nbd.2017.04.004 28400134

[B82] Rosen D. R. Siddique T. Patterson D. Figlewicz D. A. Sapp P. Hentati A.. (1993). Mutations in Cu/Zn superoxide dismutase gene are associated with familial amyotrophic lateral sclerosis. Nature 362, 59–62. 10.1038/362059a0 8446170

[B83] Runz H. Rietdorf J. Tomic I. De Bernard M. Beyreuther K. Pepperkok R.. (2002). Inhibition of intracellular cholesterol transport alters presenilin localization and amyloid precursor protein processing in neuronal cells. J. Neurosci. 22, 1679–1689. 10.1523/JNEUROSCI.22-05-01679.2002 11880497 PMC6758870

[B84] Sáiz-Vazquez O. Puente-Martinez A. Ubillos-Landa S. Pacheco-Bonrostro J. Santabarbara J. (2020). Cholesterol and Alzheimer’s disease risk: a meta-meta-analysis. Brain Sci. 10:386. 10.3390/brainsci10060386 32570800 PMC7349210

[B85] Samant N. P. Gupta G. L. (2021). Novel therapeutic strategies for Alzheimer’s disease targeting brain cholesterol homeostasis. Eur J. Neurosci. 53, 673–686. 10.1111/ejn.14949 32852876

[B86] Saunders A. M. Strittmatter W. J. Schmechel D. George-Hyslop P. H. Pericak-Vance M. A. Joo S. H.. (1993). Association of apolipoprotein E allele epsilon 4 with late-onset familial and sporadic Alzheimer’s disease. Neurology 43, 1467–1472. 10.1212/wnl.43.8.1467 8350998

[B87] Savelieff M. G. Nam G. Kang J. Lee H. J. Lee M. Lim M. H. (2019). Development of multifunctional molecules as potential therapeutic candidates for Alzheimer’s disease, Parkinson’s disease and amyotrophic lateral sclerosis in the last decade. Chem. Rev. 119, 1221–1322. 10.1021/acs.chemrev.8b00138 30095897

[B88] Scanu A. Sinisi C. Mancini M. Schiano S. (1955). [Cholesterol and lipoproteins in the humoral picture of senile and atherosclerotic dementia]. Osp. Psichiatr. 23, 183–194. 13297496

[B89] Schimanski L. A. Alperin J. P. (2018). The evaluation of scholarship in academic promotion and tenure processes: past, present, and future. F1000Res. 7, 1605–1605. 10.12688/f1000research.16493.1 30647909 PMC6325612

[B90] Schmitt S. Castelvetri L. C. Simons M. (2015). Metabolism and functions of lipids in myelin. Biochim. Biophys. Acta 1851, 999–1005. 10.1016/j.bbalip.2014.12.016 25542507

[B91] Schwartz J. L. Jones K. L. Yeo G. W. (2021). Repeat RNA expansion disorders of the nervous system: post-transcriptional mechanisms and therapeutic strategies. Crit. Rev Biochem. Mol. Biol. 56, 31–53. 10.1080/10409238.2020.1841726 33172304 PMC8192115

[B92] Segatto M. Tonini C. Pfrieger F. W. Trezza V. Pallottini V. (2019). Loss of mevalonate/cholesterol homeostasis in the brain: a focus on autism spectrum disorder and rett syndrome. Int. J. Mol. Sci. 20:3317. 10.3390/ijms20133317 31284522 PMC6651320

[B93] Simons M. Keller P. De Strooper B. Beyreuther K. Dotti C. G. Simons K. (1998). Cholesterol depletion inhibits the generation of β-amyloid in hippocampal neurons. Proc. Natl. Acad. Sci. U S A 95, 6460–6464. 10.1073/pnas.95.11.6460 9600988 PMC27798

[B94] Sipione S. Rigamonti D. Valenza M. Zuccato C. Conti L. Pritchard J.. (2002). Early transcriptional profiles in huntingtin-inducible striatal cells by microarray analyses. Hum. Mol. Genet. 11, 1953–1965. 10.1093/hmg/11.17.1953 12165557

[B200] Smalheiser N. R. Torvik V. I. (2009). Author name disambiguation. Annu. Rev. Inf. Sci. Technol. 43, 287–313. 10.1002/aris.2009.144043011320072710

[B95] Strittmatter W. J. Saunders A. M. Schmechel D. Pericak-Vance M. Enghild J. Salvesen G. S.. (1993). Apolipoprotein E: high-avidity binding to β-amyloid and increased frequency of type 4 allele in late-onset familial Alzheimer disease. Proc. Natl. Acad. Sci. U S A 90, 1977–1981. 10.1073/pnas.90.5.1977 8446617 PMC46003

[B96] Tall A. R. Yvan-Charvet L. (2015). Cholesterol, inflammation and innate immunity. Nat. Rev. Immunol. 15, 104–116. 10.1038/nri3793 25614320 PMC4669071

[B97] Testa G. Rossin D. Poli G. Biasi F. Leonarduzzi G. (2018). Implication of oxysterols in chronic inflammatory human diseases. Biochimie 153, 220–231. 10.1016/j.biochi.2018.06.006 29894701

[B98] The Huntington’s Disease Collaborative Research Group. (1993). A novel gene containing a trinucleotide repeat that is expanded and unstable on Huntington’s disease chromosomes. The Huntington’s disease collaborative research group. Cell 72, 971–983. 10.1016/0092-8674(93)90585-e 8458085

[B99] Valenza M. Rigamonti D. Goffredo D. Zuccato C. Fenu S. Jamot L.. (2005). Dysfunction of the cholesterol biosynthetic pathway in Huntington’s disease. J. Neurosci. 25, 9932–9939. 10.1523/JNEUROSCI.3355-05.2005 16251441 PMC6725556

[B100] Vanier M. T. (2010). Niemann-Pick disease type C. Orphanet. J. Rare Dis. 5:16. 10.1186/1750-1172-5-16 20525256 PMC2902432

[B101] Voet S. Prinz M. Van Loo G. (2019). Microglia in central nervous system inflammation and multiple sclerosis pathology. Trends Mol. Med. 25, 112–123. 10.1016/j.molmed.2018.11.005 30578090

[B102] Wahrle S. Das P. Nyborg A. C. Mclendon C. Shoji M. Kawarabayashi T.. (2002). Cholesterol-dependent γ-secretase activity in buoyant cholesterol-rich membrane microdomains. Neurobiol. Dis. 9, 11–23. 10.1006/nbdi.2001.0470 11848681

[B103] Wang Y. Yutuc E. Griffiths W. J. (2021). Neuro-oxysterols and neuro-sterols as ligands to nuclear receptors, GPCRs, ligand-gated ion channels and other protein receptors. Br. J. Pharmacol. 178, 3176–3193. 10.1111/bph.15191 32621622

[B104] Wolozin B. Kellman W. Ruosseau P. Celesia G. G. Siegel G. (2000). Decreased prevalence of Alzheimer disease associated with 3-hydroxy-3-methyglutaryl coenzyme A reductase inhibitors. Arch. Neurol. 57, 1439–1443. 10.1001/archneur.57.10.1439 11030795

[B105] Yeagle P. L. (1985). Cholesterol and the cell membrane. Biochim. Biophys. Acta 822, 267–287. 10.1016/0304-4157(85)90011-5 3904832

[B106] Yoon H. Flores L. F. Kim J. (2016). MicroRNAs in brain cholesterol metabolism and their implications for Alzheimer’s disease. Biochim. Biophys. Acta 1861, 2139–2147. 10.1016/j.bbalip.2016.04.020 27155217 PMC5097035

[B107] Zarrouk A. Debbabi M. Bezine M. Karym E. M. Badreddine A. Rouaud O.. (2018). Lipid biomarkers in Alzheimer’s disease. Curr. Alzheimer Res. 15, 303–312. 10.2174/1567205014666170505101426 28474568

[B108] Zarrouk A. Vejux A. Mackrill J. O’callaghan Y. Hammami M. O’brien N.. (2014). Involvement of oxysterols in age-related diseases and ageing processes. Ageing Res. Rev. 18, 148–162. 10.1016/j.arr.2014.09.006 25305550

[B109] Zhang J. Liu Q. (2015). Cholesterol metabolism and homeostasis in the brain. Protein Cell 6, 254–264. 10.1007/s13238-014-0131-3 25682154 PMC4383754

[B110] Zhang S. Zhao D. Jia W. Wang Y. Liang H. Liu L.. (2020). A bibliometric analysis and review of recent researches on TRPM7. Channels 14, 203–215. 10.1080/19336950.2020.1788355 32643506 PMC7515573

